# Supplementary material for: Detection of coinfection with Primate Erythroparvovirus 1 and arboviruses (DENV, CHIKV and ZIKV) in individuals with acute febrile illness in the state of Rio Grande do Norte in 2016
Source: PLoS Negl Trop Dis. 2023 Nov 2;17(11):e0011701. doi: 10.1371/journal.pntd.0011701 (PMC10645361; doi:10.1371/journal.pntd.0011701)
Supplement: Attachment — Submitted filename: Response to reviewer English Revised.pdf [file pntd.0011701.s001.pdf]

Institute of Tropical Medicine

University of Sao Paulo, Medical School

Av. Dr. Eneas de Carvalho Aguiar, 470

São Paulo, 05403-000

Brazil/ SP

Dr. Gregory Gromowski

Academic Editor

PLOS Neglected Tropical Diseases

April 04, 2023

Manuscript number: PNTD-D-22-01463

Dear Dr. Gregory Gromowski,

We were grateful to receive your invitation to rewrite and resubmit our article in your response of February 9,2023. Considering this, we would like to submit the enclosed revised paper, “Diagnostics and Molecular Characterization during an outbreak of Chikungunya virus and Primate Erythroparvovirus 1 in individuals with acute febrile illness in Rio Grande do Norte, Northeast of Brazil in 2016”, Manuscript PNTD-D-22-01463, for reconsideration for publication in PLOS Neglected Tropical Diseases. Please note that the revised title is “Detection of coinfection with Primate Erythroparvovirus1 and arboviruses (DENV, CHIKV and ZIKV) in individuals with acute febrile illness in the state of Rio Grande do Norte in 2016”.

We appreciate the time and work you and the reviewers have put into reading our manuscript. The feedback has been very helpful in improving the paper’s collective appearance, coherence, and content. The entire manuscript has now undergone expert editing as per your request. (Please see the enclosed editing certificate). We have revised our manuscript according to all the reviewers’ comments. The changes are highlighted in the attached manuscript, and our point-by-point responses are given in italics after the word “Response:” below.

***Responses to Reviewer’s comments:***

**Reviewer #1**

1. Comment: In this draft, entitled “Diagnostics and Molecular Characterization during an outbreak of Chikungunya virus and Primate Erythroparvovirus 1 in individuals with

acute febrile illness in Rio Grande do Norte, Northeast of Brazil in 2016", authors describe epidemiological data regarding the urban arbovirus Dengue, Zika and Chikungunya; however, only results of molecular diagnosis are described, and further characterization are missing. This is more a risk associated study, and authors should reconsider changing the title, or include more data about the viruses studied.

**Response:** *We have made the title change as suggested by the reviewer:*

*Line 1: "Detection of coinfection with Primate Erythroparvovirus1 and arboviruses (DENV, CHIKV and ZIKV) in individuals with acute febrile illness in the state of Rio Grande do Norte in 2016."*

2. Comment: Most tables are difficult to understand, as they are in Portuguese. Categories in columns must be redone. Please, also put maps with the same size and the same scale size. Check figure 6. Also, in figure 6, inform which map corresponds to DENV, CHIKV and ZIKV. Also, was B19 associated with any malformation? this information is poorly described.

**Response:** *We apologize for any parts that may have been left untranslated. We have made corrections to the categories in the tables and standardized the maps.*

*Regarding figure 6, we identified duplication of the maps, however, upon further evaluation of the context of our analysis, we removed this information from the manuscript. It was not possible to associate B19V with cases of malformations because we did not have access to the epidemiological data of individuals who tested positive.*

3. Comment: The authors conclude that more tests should be performed, which is largely known for many Brazilian states. Also, limitations regarding risk analysis based on probable cases should also be considered, as only a few samples were sent for diagnosis by PCR.

**Response:** *We agree with the reviewer and the conclusion of the study has been reformulated to reflect the percentage of samples with positive results for one or more pathogens using only the RT-qPCR and real-time PCR methodology. Based on these results, we conclude that it is necessary to expand surveillance in order to identify other possible pathogens that may be causing concurrent outbreaks with arboviruses.*

**Reviewer #2**

In this article, Vanessa dos Santos Morais et al. described a retrospective analysis of sera from acute febrile illness during year 2016 the time of CHIKV and ZIKV epidemic. Differential diagnoses were performed by PCR for CHIKV, ZIKV, Dengue and surprisingly for other viruses with generic system to flavivirus, alphaviruses enterovirus and parvovirus B19.

1. Comment: The abstract corresponds to a single descriptive analysis and a short background about the reason that led to analyse the prevalence of B19V was not presented except in the conclusion. This might be of particular interest considering the fact that Brazil was the country in which the level of fetal microcephaly was 10 or 20 times upper than the one found in the other countries. following the ZIKV epidemic that followed the CHIKV epidemic. Thus, close to 6 years later, the question remained open about the cause to such a so high defect. This point should be clarified taking in account the result obtained.

**Response:** ***Line 45:*** *For individuals presenting with acute febrile illness suspected of being infected with DENV, CHIKV or ZIKV and with negative molecular diagnosis, the Brazilian Ministry of Health recommends conducting differential diagnosis to investigate other possible pathogens that can commonly be confused with arboviruses. Among these pathogens, B19V is one of the main agents of febrile and exanthematous disease and, therefore, one of the main suspects for acute fever without etiology.*

2. Comment: To note BV19 is a DNA virus thus the diagnosis procedure is not clear here. In general, the procedure description deserved to be completed and the single reference to previous published article is not sufficient.

**Response:** ***Line 241:*** *As noted by the reviewer, B19V is a DNA virus, and therefore, the QIAmp MinElute Virus Spin Kit (Qiagen) was used, which simultaneously purifies DNA and RNA*

3. Comment: IN addition, the first part of the results was the description of the arbovirus epidemic that take in account the 86 628 cases of putative infections. This must be indicated in the abstract and explain the choice of the molecularly analyzed 713 sera.

**Response:** ***Line 52:*** *The total number of cases of arbovirus infections was described based on data from the Notifiable Diseases Information System and refers to all suspected cases reported in the state of Rio Grande do Norte. During the study period, 12,163 samples were sent to LACEN-NATAL, of which 126 were sent for molecular diagnosis of DENV, 77 were sent for molecular diagnosis of CHIKV, and 510 were sent for molecular diagnosis of ZIKV, totaling 713 samples with negative molecular results for the request viruses. It is important to note that molecular diagnosis performed by LACEN-NATAL was not simultaneous for the three arboviruses with the main circulation that year, with suspected ZIKV cases being the main focus of investigation. Therefore, upon receiving the 713 samples in our laboratory, new molecular testing was performed using*

*Multiplex Molecular Kits (which had not yet been made available in 2016) provided by the Brazilian Ministry of Health, which allow for detection of samples truly positive for the three arboviruses in question.*

**4.** Comment: In the method the available volume must be indicated if we take in account the numbers of performed PCR and extraction. At the minima, there were two extractions one for the RNA viruses the other for the DNA viruses.

**Response:** ***Line 242:*** *The eluate volume available for testing was 60µl, with 10µl used for the multiplex test, 5µl for each of the Pan Flavivirus, Pan Alphavirus, Pan Enterovirus tests, and for B19V diagnosis. Samples that tested positive were extracted and confirmed again.*

**5.** Comment: In addition, despite a very large statistical analysis the author misses to explain the relationship between the molecular analyzed samples and the general positive samples.

**Response:** *The 713 study samples were identified and molecularly analyzed. Due to lack of access to epidemiological data, it was not possible to evaluate the relationship between the positive samples overall.*

**6.** Comment: The presentation of prematurity data in pregnant women is not explained!!

**Response:** *The data on preterm birth analysis was excluded from the manuscript due to the lack of correlation with B19V cases.*

**7.** Comment: In table 1 and 2 there was a mix between English and Portuguese language.

**Response:** ***Line 305:*** *We agree with the reviewer, and the correction has been made in all tables to ensure consistency in language, using English throughout.*

**8.** Comment: There was a large lack of structure in this article.

**Response:** *We agree with the reviewer's feedback, and we have made the necessary corrections as suggested and improved the coherence and flow of content.*

**9.** Comment: The article should be largely rewritten to provide an article interesting to be read and to highlight the obtained results.

**Response:** Thank you. *We agree with this feedback, and we have made efforts to improve the article overall.*

**10.** Comment: The article should be largely rewritten to provide an article interesting to be read and to highlight the obtained results.

In the current form there is a large lack of interest and scientific proof of the obtained results as the molecular methods were poorly described.

**Response:** *We agree with the reviewer's feedback, and we have made efforts to improve description of our methodology and research findings overall.*

**Reviewer #3**

See general comment

Detailed comments:

1. Comment: Page 7, line 209: delete bracket with virus list as this is already listed in the cited reference Patel et al. Rephrase to:…for the detection of Flaviviruses, Alphaviruses and Enteroviruses as described in Patel et al., Giry et al., and Hymas et al., respectively.
Page9, line 274: ...on the condition...

**Response:** *We agree with the reviewer's feedback, and we have made the necessary corrections as suggested.*

2. Comment: Table 1 & 2 column 1: please translate to English, add legend explaining the %value in the brackets in columns 2 & 3

**Response:** *We agree with the reviewer's feedback, and we have made the necessary corrections as suggested.*

3. Comment: Page 11, line 327: Here you are talking about the disease not the virus and if the value sin in table 1 are correct its actually:
"Higher among Chikungunya suspects and lower among Dengue and Zika fever suspects."

**Response:** *We agree with the reviewer's feedback, and we have made the necessary corrections in line 289.*

4. Comment: Page 11, line 336: Please rephrase - unclear to me.

**Response:** *We agree with the reviewer's feedback, and we have made the necessary corrections in line 299.*

5. Comment: Page 13, line 349: Regarding arbovirus infections of pregnant women, 2,640 cases were
considered probable in the SINAN database, 1.8% for DENV, 4.0% for CHIKV and 15.9% for ZIKV respectively and the highest percentage was observed in the age groups between 20-29 years, being 50.3% for CHIKV, 50.1% 354 for DENV and 51.2% for ZIKV (Table 2).

**Response:** *We agree with the reviewer's feedback, and we have made the necessary corrections.*

6. Comment: Page 17, line 387: of which samples? ...all samples or samples from pregnant women? Which group do the n= 492 represent?

**Response:** *We agree with the reviewer's feedback, and we have made the necessary corrections as suggested in page 324, line 324.*

7. Comment: Page 17, line 389: List First double arbovirus infections, then arbovirus +B19, then Doble arbovirus +B19 infections Percentage is missing for CHIKV+B19.

**Response:** *We agree with the reviewer's feedback, and we have made the necessary corrections as suggested.*

8. Comment: Page21, line 464. and ZIKV infection.... Something is missing in the sentence please rephrase.

**Response:** *The data on preterm birth analysis was excluded from the manuscript due to the lack of correlation with B19V cases.*

9. Comment: page 25, line 559: If These figures are the same as on page 17 line 387 then please heck the percentage for CHIKV & DENV in both paragraphs.

**Response:** *We agree with the reviewer's feedback, and we have made the necessary corrections as suggested.*

10. Comment: Page 25, line 566: Why did you test for B19? Because of the issue put know it can cause in pregnancy or because of DiPaola`s study. There is no problem in clearly saying well we saw DiPaola´s study and we wanted to include these analyses to see if we could falsify / confirm what was described.

**Response:** *As per the recommendations of the Ministry of Health when testing for the three main arboviruses yields negative results, further investigation is conducted for other pathogens, including B19V. The study by Di Paola showed similar findings, as the cohort under investigation consisted of individuals suspected of arbovirus infection.*

11. Comment: Altogether this is an interesting study worth publishing. However, there are some structural problems with the paper and some language issues.

    The motivation to look for premature births is not well explained in the Introduction and Results. The first mention of the effect ZIKV on pregnancy being the driver for the study analyses is in the discussion in line 555.

    The Introduction should set out the motivation and hypotheses that determined the design of the study based on existing literature. The motivation should be referred to in the appropriate sections of the results.

The discussion should be sharpened and explain how the results confirm or dispel the hypothesis.

**Response:** *We agree with the reviewer's suggestions and have made the necessary corrections.*

All authors have read and approved the changes made to the manuscript. We hope that the revised paper is now suitable for inclusion in PLOS Neglected Tropical Diseases, and we look forward to hearing from you.

Yours sincerely,

Vanessa S. Morais
Doctorate Student of Institute of Tropical Medicine
University of Sao Paulo, Medical School
Av. Dr. Eneas de Carvalho Aguiar, 470 – Predio 2 - 1° andar
Cerqueira César – 05403-000 – São Paulo – SP – Brazil
Tel: +55 11 3061-7041/ 3061-7042
Cel: +55 11 3011-4937
va.morais@usp.br
