# Supplementary material for: Detection of coinfection with Primate Erythroparvovirus 1 and arboviruses (DENV, CHIKV and ZIKV) in individuals with acute febrile illness in the state of Rio Grande do Norte in 2016
Source: PLoS Negl Trop Dis. 2023 Nov 2;17(11):e0011701. doi: 10.1371/journal.pntd.0011701 (PMC10645361; doi:10.1371/journal.pntd.0011701)
Supplement: Attachment — Submitted filename: Response to reviewer.docx [file pntd.0011701.s002.docx]

Institute of Tropical Medicine

University of Sao Paulo, Medical School

Av. Dr. Eneas de Carvalho Aguiar, 470

São Paulo, 05403-000

Brazil/ SP

Dr. Gregory Gromowski

Academic Editor

PLOS Neglected Tropical Diseases

August 20, 2023

Manuscript number: PNTD-D-22-01463R1

Dear Dr. Gregory Gromowski,

We were grateful to receive your invitation to rewrite and resubmit our article in your response of July 27, 2023. Considering this, we would like to submit the enclosed revised paper, “Detection of coinfection with Primate Erythroparvovirus 1 and arboviruses (DENV, CHIKV and ZIKV) in individuals with acute febrile illness in the state of Rio Grande do Norte in 2016”, Manuscript PNTD-D-22-01463R1, for consideration for publication in PLOS Neglected Tropical Diseases.

We appreciate the time and work you and the reviewers have put into reading our manuscript. The feedback has been very helpful in improving the paper’s collective appearance, coherence, and content. The entire manuscript has now undergone expert editing as per your request. (Please see the enclosed editing certificate). We have revised our manuscript according to all the reviewers’ comments. The changes are highlighted in the attached manuscript, and our point-by-point responses are given in italics after the word “Response:” below.

***Responses to Reviewer’s comments:***

**Reviewer #2**

Methods

-Are the objectives of the study clearly articulated with a clear testable hypothesis stated?

-Is the study design appropriate to address the stated objectives?
-Is the population clearly described and appropriate for the hypothesis being tested?
-Is the sample size sufficient to ensure adequate power to address the hypothesis being tested?
-Were correct statistical analysis used to support conclusions?
-Are there concerns about ethical or regulatory requirements being met?

1. Comment: The objective of the study is more or less clearly presented and should be highlighted in the abstract and at the end of the introduction or background. To note the abstract is quite too long.
   The study design however is appropriate, but the presentation of the samples tested in this study is not clear. In fact what we understand is that the 713 samples were NOT tested by molecular method before the present study and not that they were negative by a molecular test before to be send to lab.
   So the population is finally not well described in the abstract (even if many details were given in the results)
   To note this is not clear who performed the molecular test and a major weakness of the paper is the lack of details of this molecular test both the one that give positive results and those that were negative. Details (materials, primers, positive and negative controls must be given).
   Finaly there was no clear hypothesis thus I cannot conclude about the sample size to test it.

**Response:** *Between January and August 2016, the Notifiable Diseases Information System (SINAN) received a total of 86,628 notifications of suspected arbovirus cases in the State of Rio Grande do Norte, with 12,163 samples sent to LACEN-NATAL. Among these, 713 samples from individuals with suspected acute febrile illness due to arbovirus were forwarded for molecular diagnosis, with 126 samples tested for DENV, 77 samples tested for CHIKV, and 510 samples tested for ZIKV. The molecular tests conducted by LACEN-NATAL for the respective arboviruses yielded negative results, and the samples were subsequently sent to the Laboratory of Medical Parasitology at the Institute of Tropical Medicine of Sao Paulo. We received a total of 713 serum samples, comprising 667 women, of which 354 were pregnant, and 46 men. The initial screening was performed through molecular analysis using the Multiplex RT-qPCR methodology for the concurrent detection of DENV, CHIKV and ZIKV. For the differential diagnosis, other viruses from the Flavivirus, Alphavirus, Enterovirus genera, and the Primate Erythroparvovirus 1 species, previously known as Parvovirus B19 (B19V), were included, following the guidelines established by the Ministry of Health.*

2. The choice of the viruses tested (Flavivirus, alphavirus, enterovirus or BV19) MUST be explained in the introduction and not only in the discussion.

**Response:**  *We agree with this feedback, and we have made efforts to improve the article overall.*

***Line 175:*** *Our study describes the laboratory results of individuals with acute febrile syndromes reported with suspected arboviral diseases to the Notifiable Diseases Information System (SINAN), during the period from January to August 2016. We emphasize the emergence and reemergence of arboviral diseases, particularly in regions with concurrent circulation. Additionally, we demonstrate molecular approaches that contribute to a rapid and accurate differential diagnosis of DENV, CHIKV and ZIKV, as well as their implications for other viruses of the Flavivirus, Alphavirus and Enterovirus genera and for the Primate Erythroparvovirus 1 (B19V) species of the Erythroparvovirus genus.*

3. I imagine that the hypothesis that support this paper is “as the patient are negative for the test, it might be another pathogen (virus?) that induced the disease”.
   Here the statistical test question is not appropriate.
   The ethical and regulatory requirement are meet in this article.

**Response:**  *The hypothesis underlying this article suggests that other viruses, in addition to arboviruses, may be circulating concomitantly and causing silent epidemics, as evidenced by the case of B19V described in this study. Furthermore, we emphasize the importance of differential diagnosis, even in samples with previously established positive results. Through this approach, it was possible to detect coinfections and make significant contributions to the surveillance of arboviruses and other viruses associated with acute febrile syndromes.*

Conclusions

-Are the conclusions supported by the data presented?

-Are the limitations of analysis clearly described?

-Do the authors discuss how these data can be helpful to advance our understanding of the topic under study?

-Is public health relevance addressed?

1. Comment: The conclusion is supported by the data but the reason to the choice of the virus tested is not clearly assessed and must be given in the introduction after the background and moreover the methods used fully described with positive and negative control, cut-off of positive response, volume of the samples used and is there are whole blood, blood spot on paper, sera etc.

The limitations are clearly described as well as the usage of them for knowledge advance. BUT one more time the methods to obtain the result are not discussed.
Finally, the public health relevance is addressed.

**Response:** *We agree with this feedback, and we have made efforts to improve the article overall.*

***Line 244:*** *The total amounts of nucleic acids were extracted from 200ul aliquots of human serum samples using the QIAamp MinElute Virus Spin Kit (Qiagen), following the manufacturer’s instructions. The eluate containing 60ul of total RNA and DNA was stored in a -80°C freezer until ready for use. For the RT-qPCR assay, we used the Kit Molecular ZDC – BioManguinhos (Instituto de Tecnologia em Imunobiológicos, Fundação Oswaldo Cruz – RJ – Brasil). Reverse transcription of RNA into cDNA preceded the amplification. The target-specific amplification methodology using fluorescence-labeled probes was employed to determine the presence, discriminative for DENV, CHIKV and ZIKV. The Molecular Kit was standardized to detect truly positive DENV samples with Ct values less than or equal to 33, and for truly positive CHIKV and ZIKV samples with Ct values less than or equal to 38. In all reactions, the negative and positive controls provided in the Kit were used. All samples were initially tested simultaneously for DENV, CHIKV and ZIKV, followed by differential diagnosis.*

*For Flavivirus detection we followed the protocol established by Patel et al. (10). Cell culture samples of the four DENV serotypes and Saint Louis Encephalitis virus (SLEV) were used as positive controls for this assay. For Alphavirus detection we followed the protocols established by Giry et al. (11). Cell culture samples of Mayaro virus (MAYV) and CHIKV were used as positive controls for this assay. For Enterovirus detection we followed the protocol established by Hymas et al. (12). Positive controls used were derived from cell culture samples of Echovirus 30. For B19V species detection, the protocol of Jia J et al. (13). Was followed. Positive controls used in this assay were obtained from known positive samples stored in the laboratory. Th processing of all samples included negative controls as well as internal controls to ensure the reliability of the reaction. The reactions were conducted using the ABI 7500 real-time PCR system.*

Editorial and Data Presentation Modifications?

Use this section for editorial suggestions as well as relatively minor modifications of existing data that would enhance clarity. If the only modifications needed are minor and/or editorial, you may wish to recommend “Minor Revision” or “Accept”.

1. The abstract is too long, and the thru objective need to be clearly stated

**Response:** *We agree with this feedback, and we have made efforts to improve the article overall.*

Summary and General Comments

Use this section to provide overall comments, discuss strengths/weaknesses of the study, novelty, significance, general execution and scholarship. You may also include additional comments for the author, including concerns about dual publication, research ethics, or publication ethics. If requesting major revision, please articulate the new experiments that are needed.

Detection of coinfection with Primate Erythroparvovirus 1 and arboviruses (DENV, CHIKV and ZIKV) in individuals with acute febrile illness in the state of Rio Grande do Norte in 2016.

1. In this new version of their article, Vanessa dos Santos Morais et al. answered to most of the questions and take in account the main suggestions. However, the description of the molecular analysis remained very poor. In the minima, the fact that the methods used were real time PCR should be given and not nested classicals. Even if the methods are already published some details about the targeted gene region, the usage of multiplex system etc… deserved also to be given. We know very well that most of the methods needs to be adapted or tested within a lab. Thus a short schapter explaining the quality control and the positive and negative control used as well as the cut-off chosen to consider a Ct as significant to give a positive result (i.e. detection of the DNA or RNA target) must be provided.
   Thus mainly because all the paper conclusion is based on the molecular test value and quality….I cannot support the publication in the current form

**Response:** *We agree with this feedback, and we have made efforts to improve the article overall.*

2. Minor comments:

The abstract is too long; this is not an abstract but a copy past of some section of the paper.

**Response:** *We agree with this feedback, and we have made efforts to improve the article overall.*

*Line 26: The correction was made objectively, meeting the criteria of the journal, as well as incorporating the previous suggestions from the reviewers.*

3. Line 172-173: this is not common to described in increase of 2016 compared to 2017! This must be a decrease of 2017 compared to 2016. The time direction is from the past to the future (We are not in “return to the future” movies).

**Response:** *I apologize for the incorrect phrasing of the sentence. The correction was made by indicating the correct year for comparison.*

**Reviewer #3**

The paper is much improved. The following minor edits are still required italics only if you use the taxonomic designation not if you generalize!

1. page 8, line 247   .... of Flaviviruses, Alphaviruses and Enteroviruses.

**Response:** *We agree with this feedback, and we have made efforts to improve the article overall.*

2. page 13, line 360   ... for other Flaviviruses, Alphaviruses and Enteroviruses showed negative results...

**Response*:***

***Line 362:*** *The 713 individuals subjected to investigation within the Flavivirus and Alphavirus genera presented outcomes that align with those obtained in the Multiplex ZDC – BioManguinhos assay (unshown data). In contrast, the results observed for the Enterovirus genus were negative.*

3. page 16, line 445   ...After identifying DENV, CHIKV and ZIKV, we proceeded to test for other Flaviviruses, Alphaviruses and Enteroviruses, as well as for B19V...

**Response:** *We agree with this feedback, and we have made efforts to improve the article overall.*

4. page 16, line 447   ...In our analysis, there were no additional identifications of Flaviviruses and Alphaviruses other than DENV, ZIKV and CHIKV.

**Response:** *We agree with this feedback, and we have made efforts to improve the article overall.*

5. page 12, line 330   ... you chose not to use the suggested phrasing of the first review which was:
   Regarding arbovirus infections of pregnant women, 2,640 cases were considered probable in the SINAN database, 1.8% for DENV, 4.0% for CHIKV and 15.9% for ZIKV respectively and the highest percentage was observed in the age groups between 20-29 years, being 50.3% for CHIKV, 50.1% 354 for DENV and 51.2% for ZIKV (Table 2). You knew paragraph however is again not well written and I suggest you use the suggested paragraph above after all or ask any other native speaker to help you rephrase the paragraph which cannot stay as it is.

**Response:** *We agree with this feedback, and we have made efforts to improve the article overall.*

*Line 364: In the context of pregnant women, a total of 2,640 suspected cases of arboviral infections were recorded in the SINAN database, of which 38% were associated with DENV, 40.5% with CHIKV and 21.7% with ZIKV. Regardin suspected cases of DENV, CHIKV and ZIKV, the age grouo with the highest observed percentage was between 20 and 29 years, totaling 50.3% for suspected cases of CHIKV, 50.1% for DENV and 51.2% for ZIKV (Table 2).*

6. page 13, line 355   ... showed positivity

**Response:** *We agree with this feedback, and we have made efforts to improve the article overall.*

All authors have read and approved the changes made to the manuscript. We hope that the revised paper is now suitable for inclusion in PLOS Neglected Tropical Diseases, and we look forward to hearing from you.

Yours sincerely,

Vanessa S. Morais

Doctorate Student of Institute of Tropical Medicine

University of Sao Paulo, Medical School

Av. Dr. Eneas de Carvalho Aguiar, 470 – Predio 2 - 1° andar

Cerqueira César – 05403-000 – São Paulo – SP – Brazil

Tel: +55 11 3061-7041/ 3061-7042

Cel: +55 11 3011-4937

va.morais@usp.br
